# Compensation method for respiratory motion in proton treatment planning for mobile liver cancer

**DOI:** 10.1120/jacmp.v14i2.4055

**Published:** 2013-03-04

**Authors:** Hojin Jeong, Se Byeong Lee, Seung Hoon Yoo, Young Kyung Lim, Tae Hyun Kim, Seyjoon Park, Gyu Young Chai, Ki Mun Kang, Dongho Shin

**Affiliations:** ^1^ Proton Therapy Center, Research Institute and Hospital National Cancer Center Goyang Korea; ^2^ Department of Radiation Oncology Institute of Health Science, Gyeongsang National University Hospital JinJu Korea; ^3^ Department of Radiation Oncology Cha Bundang Medical Center, Cha University Seongnam Korea

**Keywords:** proton therapy, four‐dimensional computed tomography, four‐dimensional proton plan, respiration motion, field‐specific proton margin

## Abstract

We evaluated the dosimetric effect of a respiration motion, and sought an effective planning strategy to compensate the motion using four‐dimensional computed tomography (4D CT) dataset of seven selected liver patients. For each patient, we constructed four different proton plans based on: (1) average (AVG) CT, (2) maximum‐intensity projection (MIP) CT, (3) AVG CT with density override of tumor volume (OVR), and (4) AVG CT with field‐specific proton margin which was determined by the range difference between AVG and MIP plans (mAVG). The overall effectiveness of each planning strategy was evaluated by calculating the cumulative dose distribution over an entire breathing cycle. We observed clear differences between AV G and MIP CT‐based plans, with significant underdosages at expiratory and inspiratory phases, respectively. Only the mAVG planning strategy was fully successful as the field‐specific proton margin applied in the planning strategy complemented both the limitations of AVG and MIP CT‐based strategies. These results demonstrated that respiration motion induced significant changes in dose distribution of 3D proton plans for mobile liver cancer and the changes can be effectively compensated by applying field‐specific proton margin to each proton field.

PACS numbers: 87.55.D; 87.53.Bn; 87.53.Jw; 87.55.dk

## I. INTRODUCTION

Proton therapy has dosimetric advantages relative to conventional photon and electron therapies. These advantages are due primarily to the depth‐dose characteristics of protons, in which proton dose gradually increases until the Bragg‐peak position and decreases abruptly thereafter.^(^ ^1^ ^)^ The advantages of proton therapy include a reduced integral dose due to a lower entrance dose relative to that in photon therapy, absence of an exit dose, and a better ability to control the desired dose distribution by controlling the stopped positions of protons. Proton therapy, therefore, has real benefits to improve local control probability and sparing of normal tissue.^(^ ^2^ ^–^ ^4^ ^)^

The depth‐dose properties of protons, however, make it difficult to perform the treatments as originally planned because small changes can induce large variations. It therefore requires higher standards in treatment procedures, including imaging,^(^ ^5^ ^)^ quality assurance,^(^ ^6^ ^)^ and patient set‐up.^(^ ^7^ ^,^ ^8^ ^)^ In addition, proton therapy demands extra attention in treatment planning to reflect the actual density distribution of a patient's body since proton dose is very sensitive to internal density distributions. For example, proton dose calculations with density‐enhanced computed tomography (CT) images with contrast media, which are frequently injected into patients to accurately identify tumor volume or critical organs at risk,^(^ ^9^ ^)^ may be incorrect.^(^ ^10^ ^,^ ^11^ ^)^ Thus, all density mismatches or artifacts in planning CT scans should be corrected accordingly to their real densities. This is the same for the respiration‐induced anatomical change because it can also lead to relative change in density distribution and, consequently, to miscalculation of proton dose distribution.^(^ ^12^ ^,^ ^13^ ^)^ To date, however, a proper dose calculation method, such as a four‐dimensional dose (4D dose) calculation combined with deformable image registration,^(^ ^14^ ^,^ ^15^ ^)^ has not been developed in proton planning systems.

Several approximation methods to deal with the respiration‐induced density change have been proposed.^(^ ^12^ ^,^ ^15^ ^–^ ^18^ ^)^ Among them, the density‐override method,^(^ ^12^ ^,^ ^15^ ^)^ in which the CT density of the treatment target volume was replaced by the average density of the gross tumor volume to account for internal gross tumor motion, has been reported to be very successful in mobile lung cancer treatment. However, the effectiveness of this method has not been widely evaluated at other treatment sites.

In the present work, we quantitatively investigated the dosimetric effect of the respiration‐induced change in the internal density distribution for mobile liver cancers. We also evaluated the dosimetric effectiveness of the previously proposed methods, including density‐override method,^(^ ^12^ ^,^ ^15^ ^)^ to compensate the respiration motion, but none of them was fully successful in the present liver cancer patients. We have, therefore, proposed an alternative that uses the concept of field‐specific proton margin, which yielded better result than any of the previous models.

## II. MATERIALS AND METHODS

### A. Calculation accuracy of proton planning system

**Figure 1:**
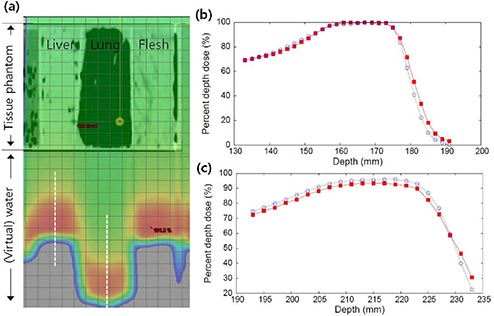
Simulated proton‐dose distribution (a) for real tissue phantoms composed of liver, lung, and flesh tissues; simulated (open circles) (b) and measured (filled squares) (c) depth‐dose curves scanned along the vertical lines below (b) liver and (c) lung tissues. The lines, along which the depth‐dose curve were scanned, are shown in (a) by white dashed lines.

All calculations for proton‐dose distribution were performed with the Eclipse proton planning system (Eclipse proton v8.1, Varian Medical Systems, Palo Alto, CA). This planning system was based on the method developed by Schneider et al.^(^ ^10^ ^,^ ^19^ ^,^ ^20^ ^)^ in which CT Hounsfield unit (HU) was converted into relative stopping power of proton to take into account the tissue inhomogeneity effect. We precisely measured the relationship between CT HU and the relative stopping power of various different tissue‐equivalent materials using a CT phantom (Electron Density phantom 062, CIRS, Norfolk, VA). This relationship was incorporated into the planning system and used as the basic input for dose calculation. Although the method described above has been well‐verified from previous studies,^(^ ^10^ ^,^ ^19^ ^,^ ^20^ ^)^ we performed actual measurements to make further clear the calculation accuracy of our proton‐planning system. We prepared a real tissue phantom composed of various organs of an animal with 10 cm depth. This tissue phantom put in the water tank during measurement with irradiation of 195 MeV proton beam. The proton dose and the range shifts of proton beam due to the presence of tissue phantom were measured by scanning the depth‐dose curves in the water. The simulation was performed under the same geometric condition with the measurement using the CT image of tissue phantom where HU values surrounding the phantom were replaced by water‐equivalent HU value (0 HU) to mimic the water tank. The results showed that the measured depth‐dose curves agreed well with the simulated one (see Fig. 1). The depth‐dose curves scanned on the lines below the liver (Fig. 1(b)) and flesh (data not shown here) tissues agreed with the calculated results within 2%/2 mm, and that measured below lung tissue (Fig. 1(c)) agreed within 4%/3 mm. These results demonstrated that our treatment planning reflected well the tissue inhomogeneity effect, and reproduced well the actual dose distribution.

### B. Patient selection

We selected seven patients with typical gross tumors located near the dome of the liver. All patients received proton therapy at the Proton Therapy Center of the National Cancer Center in Korea. We selected these patient cases because the dosimetric effects of respiration have more impact than any other sites due to strong heterogeneous and movable condition. The Hounsfield number for normal liver tissue, 100–130, is much higher than that of adjacent lung tissues (−900 to −300 HU), resulting in ∼3−4 times higher stopping power for proton in liver than in lungs. Therefore, protons would be strongly perturbed at the interface of two heterogeneous organs if the interface is largely moved during irradiation. Indeed, significant movements of liver and lung were observed on 4D CT measurements (Table 1). These motions occurred predominantly in the cranial–caudal direction, with the magnitude of peak‐to‐peak motion of the liver‐dome apex ranging from 0.5 cm to 1.3 cm, which is comparable to tumor motion in lung cancer patients.^(^ ^15^ ^)^

**Table 1:** Planned target volume (PTV) and peak‐to‐peak breathing motion for liver‐dome apex in cranial–caudal direction.

*Patents*	*PTV (cm* ^*3*^ *)*		*Volume in Lung* ^*^
	*Liver Motion (cm)*	*Total Volume*	
A	63.8	12.38	1.3
B	60.3	14.2	1.3
C	90.5	3.36	0.5
D	830.8	41.29	0.8
E	690.2	32.20	0.5
F	110.2	11.93	0.7
G	150.7	11.42	0.9

* The volume intersected with lungs.

### C. Four‐dimensional computed tomography (4D CT) and target delineation

Following shallow respiration training, a set of 4D CT scans consisting of 10 equally divided phases was obtained.^(^ ^21^ ^)^ Each phase of CT scan was named after percentage in respiration phases: the 0% phase CT scan corresponding to end‐of‐inspiration was named CT0% and the 50% phase CT scan corresponding to end‐of‐expiration was named CT50%. After acquisition of 4D CT scan set, two CT scans were reconstructed, an average (AVG) and a maximum‐intensity‐projection (MIP) scan, by taking the average and maximum CT numbers, respectively, in the 4D CT dataset at each pixel point.

**Figure 2:**
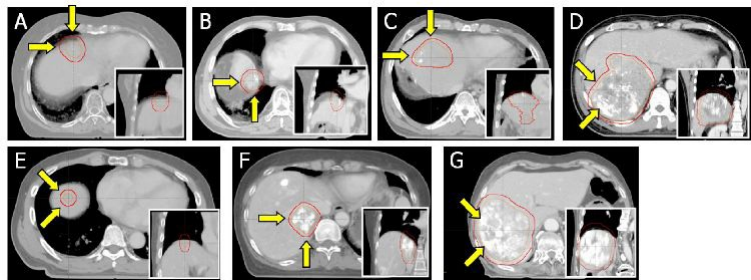
Axial views of the tumor centers on the AVG CT scans. The red contours are the PTV volume and the arrows indicate the entrance proton beam directions. The inset in each figure is the coronal view for tumor volume.

The gross tumor volume (GTV) was first contoured on each phase of 4D CT scans. And then, all the GTV drawn on every phase CT scans were merged on the AVG (MIP) CT to create a motion volume of gross tumor, called the internal tumor volume (ITV). The ITV for each patient was further expanded uniformly by the planning margin of ∼1% mm in all spherical directions, yielding a planned tumor volume (PTV). As the internal and planning margins were added in PTV, the final PTV extended to lung tissues (see Table 1 and Fig. 2). Table 1 shows the volumes of PTV intersecting with the lungs on AVG CT, as well as the entire PTV. Because, the proton plan is very sensitive to density variations in tumor volume, we derived an additional CT scan from AVG CT for each patient, where the density of PTV was overridden to the average gross tumor density; this is called OVR CT. We note here that the density override was applied to PTV, instead of ITV as done in the previous study,^(^ ^15^ ^)^ because the correction efficiency was very poor in the present patient cases if the density override was restricted to ITV.

### D. Treatment plans

All the treatment plans were designed to PTV based on a double‐scattering mode which required an aperture collimator and a range compensator for each treatment field.^(^ ^15^ ^,^ ^16^ ^,^ ^22^ ^)^ The aperture collimator was designed for the projected shape of PTV with uniformly expanded 1 cm aperture margins. The range compensator was designed to optimally fit the prescribed isodose surface to the distal surface of PTV based on calculations of simple effective path length of primary protons.^(^ ^16^ ^,^ ^22^ ^)^ In addition, a 1 cm border smoothing margin was applied in designing the range compensator. In general, an additional margin to broaden the compensator curvature (smearing margin)^(^ ^15^ ^–^ ^18^ ^,^ ^22^ ^)^ is applied to the range compensator to account for the motion of internal organs and any setup uncertainty. In this study, however, we did not apply a smearing margin because this study was aimed at quantifying the dosimetric effects of internal density variation instead of a simple correction. With the designing for the devices used in each proton field, we optimized the proton beam range and spread out Bragg peak (SOBP) width to that spread out proton beam properly covered the entire PTV, where the distal range and the spread out Bragg peak (SOBP) width were defined as the depth to distal 90% isodose line and the distance between proximal and distal 90% isodose lines, respectively.

We initially prepared three‐dimensional (3D) proton plans on the AVG, MIP, and OVR CT scans, called the AVG, MIP, and OVR plans, respectively. All treatment plans were constructed with two equally weighted coplanar proton fields which were orthogonally aligned with respect to each other to minimize the overlap between the fields (see Fig. 2 for details in field orientation). The overall dose distribution produced by the two orthogonal proton fields was normalized to have 100% PTV coverage at the 95% of the prescribed dose.

As will be described further below, all the planning strategies described above did not successfully create adequate dose coverages at every respiration phase. Therefore, we suggested an additional planning strategy in the present study. This was basically the same with the AVG planning, but the distal range for each proton field was manually increased yet keeping all other planning parameters the same with the AVG plan. The magnitude of distal range increment for each field was determined as the distal range difference between the fields in MIP and in AV G plans. This modified AVG plan is referred as mAVG plan, hereafter.

### E. Plan evaluation

In order to investigate the dosimetric change caused by respiration motion, we recalculated the dose distribution of each 3D proton plan with replacing the planning CT to one of the 4D CT scans. In this process, all planning parameters and beam modifier geometries (i.e., aperture collimators and range compensators), except for planning CT, were kept identical to those for ordinary 3D plans. We repeated the calculation over the entire 4D CT scans. The dose differences between the ordinary plan and the recalculated plan with substitution of CT image was quantitatively evaluated using the target dose indices of $D_{95\%}$, $D_{99\%}$, and $D_{100\%}$, defined as the minimal target doses to cover 100%, 99%, and 95% of target volume, respectively. We assumed that changes in the above target dose indices within 2% were acceptable. That is, if all indices of the plan were changed within ±2%, the dose change resulting from respiration motion was tolerable, whereas, if any change in the indices was out of the range, it was not. As a reference of planning quality, we also calculated the conformity index (CI) for each plan, defined as the ratio between the total irradiated volume and the target volume receiving at least the prescribed dose.

To investigate the full effect of respiration‐induced changes in dose distribution, we composed the dose distributions calculated on all 10 phases of each 4D CT scan onto a single representative CT scan. We choose the AVG CT as a representative CT scan since it is where all anatomical changes are averaged during respiration. This composed dose for each plan was referred to as a 4D dose to distinguish it from the apparent dose of the 3D plan. The respiration‐induced change in 4D dose was also evaluated with the same criteria of acceptability range of ±2% for the change in target dose indices ($D_{95\%}$, $D_{99\%}$, and $D_{100\%}$).

## III. RESULTS

### A. 3D proton plan

All 3D proton plans for each patient were designed using the same planning criteria under the same treatment mode. Thus, the AVG, MIP, and OVR plans for each patient had effectively the same dose for PTV, as summarized in Table 2. The target dose indices of $D_{95\%}$, $D_{99\%}$, and $D_{100\%}$ for each patient were the same within ∼1% and none of the CI differences among each patient's plans were higher than 5%. However, the entrance‐proton characteristics were changed with the planning strategies. The proximal and distal edges of spread out proton beams were maximal in the MIP plans, while minimal in the AV G plans in all seven patients (Table 2). The depths to the distal edges of the SOBP and the proton ranges were 0.03−0.91 cm larger in the MIP than in the AVG plans. The SOBP widths were also larger in the MIP plans by 0.03−0.34 cm than the AVG plans. The calculated proton beam ranges and SOBP widths for the OVR plans differed from those for the AVG plans, by 0.00−0.34 cm for distal ranges and by −0.33−0.34 cm for SOBP widths.

**Table 2:** Target dose, conformity index, distal range, and SOBP width of each proton plan.

						*Field 1*		*Field 2*	
*Patient*	*Plan*	$D_{95\%}\left(\%\right)$	$D_{99\%}\left(\%\right)$	$D_{100\%}\left(\%\right)$	*C.I*	*Range (cm)*	*SOBP (cm)*	*Range (cm)*	*SOBP (cm)*
A	AVG	100.00	98.43	94.90	1.55	12.37	6.84	8.84	5.68
	OVR	100.00	97.89	95.20	1.57	12.37	6.84	9.18	6.02
	MIP	100.00	98.41	95.00	1.55	13.10	6.71	9.49	6.02
B	AVG	100.00	98.91	96.40	1.31	11.40	6.56	13.21	5.49
	OVR	100.00	98.59	95.90	1.34	11.51	6.23	13.22	5.50
	MIP	100.00	97.87	95.60	1.38	11.60	6.31	13.36	5.54
C	AVG	100.00	98.28	95.00	1.47	11.92	5.88	13.41	5.28
	OVR	100.00	98.42	94.00	1.45	11.94	5.81	13.55	5.34
	MIP	100.00	98.58	94.80	1.46	12.87	5.88	14.19	5.47
D	AVG	100.00	97.75	93.90	1.09	12.24	3.73	8.40	4.03
	OVR	100.00	98.11	93.50	1.09	12.23	3.62	8.40	3.93
	MIP	100.00	98.09	93.90	1.08	13.14	3.82	9.10	3.85
E	AVG	100.00	99.31	96.37	2.01	17.37	15.48	19.88	14.83
	OVR	100.00	98.28	96.37	2.10	17.37	15.48	19.88	14.80
	MIP	100.00	98.44	96.37	2.08	17.40	15.51	20.12	14.88
F	AVG	100.00	95.00	94.70	1.08	14.96	11.54	11.81	8.60
	OVR	100.00	95.70	94.45	1.11	14.96	11.54	11.82	8.61
	MIP	100.00	95.79	94.80	1.08	15.16	11.69	11.99	8.75
G	AVG	100.00	97.58	94.80	1.22	12.63	10.85	15.41	12.62
	OVR	100.00	98.40	94.90	1.21	12.74	10.96	15.51	12.65
	MIP	100.00	97.43	94.50	1.20	12.78	10.95	15.70	12.61

CI=conformity index; $D_{x\%}=\text{minimal}$ dose of x% of target volume; SOBP=spread−out Bragg peak. Two proton fields in each plan were listed in clockwise order from the posterior direction (see Fig. 2).

### B. respiration‐induced dose changes

#### B.1 AVG plan

**Figure 3:**
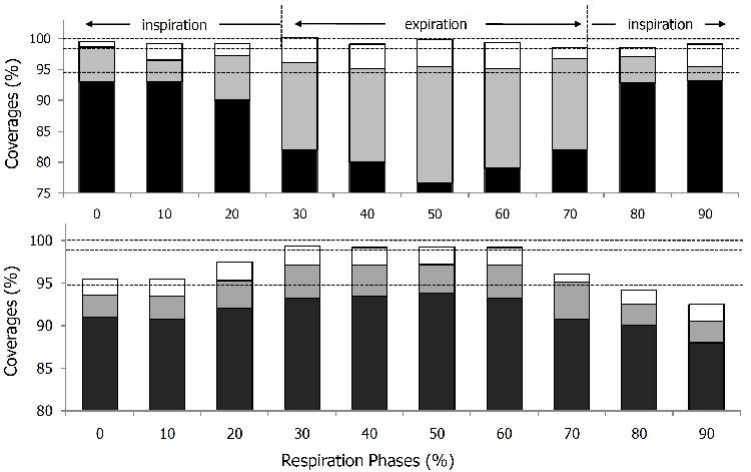
Target dose changes with respiration phase for AVG (upper) and MIP (lower) plans for patient A. The dark, gray, and white colors represent the $D_{95\%}$, $D_{99\%}$, and $D_{100\%}$ values. The corresponding values of $D_{95\%}$, $D_{99\%}$, and $D_{100\%}$ in the ordinary 3D plans are indicated, from to top to bottom, by dashed lines, respectively, for references.

**Figure 4:**
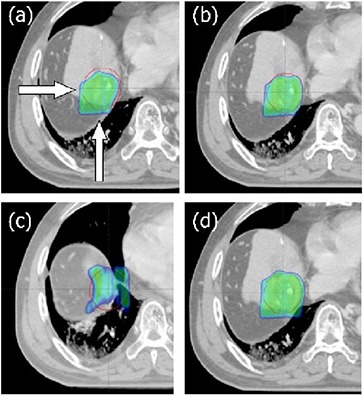
Planar dose distribution for patient A: (a) AVG plan on end‐of‐expiratory CT50%, (b) OVR plan on CT50%, (c) MIP plan on full inspiratory CT0%, and (d) mAVG plan on CT50%, where the red contours are the PTV and colorwash area are the region receiving at least 95% of the prescribed dose. The entrance proton beam directions are marked by arrows in (a).

Figure 3(a) shows the respiration‐induced change in target dose for the AVG plan of patient A, for example. Figure 3 shows that all target dose indices ($D_{100\%}$, $D_{99\%}$, and $D_{95\%}$), except for $D_{95\%}$ at 20% phase, agreed within ±2% with those in ordinary 3D plans in inspiratory (0%–20% and 80%–90%), but not in expiratory (30%–70%) phases. That is because the proton beam ranges on AVG CT were basically underestimated for expiratory CT scans having higher CT numbers than AV G CT. This resulted in systematic shifts of isodose surfaces in the expiratory phases toward the proximal direction, consequently resulted in significant underdosages at the distal side of target volume on the expiratory CT scans (see Fig. 4(a). This dose‐distribution change was common to all seven patients. As summarized in Table 3, at the end‐of‐expiratory phase, the target‐dose indices for these patients were significantly changed over the acceptable range, with decreasing ranges of 2.4%–50.1% for $D_{100\%}$, 0.6%–22.1% for $D_{99\%}$, and 0%–10.8% for $D_{95\%}$, while, at full‐inspiration phase were not significant, as the changes in all target‐dose indices ($D_{100\%}$, $D_{99\%}$, and $D_{95\%}$) were fully acceptable (<2%) in four of the seven patients, and were no higher than 4% in other three patients. These findings demonstrate that the AVG planning strategy reproduced the dose distribution in the inspiratory phases well, but was largely limited for the expiratory phases.

**Table 3:** Target doses at the full‐inspiratory and at the end‐of‐expiratory phases.

			*Full‐inspiration*			*End‐of‐expiration*	
*Patient*	*Plan*	$D_{95\%}\left(\%\right)$	$D_{99\%}\left(\%\right)$	$D_{100\%}\left(\%\right)$	$D_{95\%}\left(\%\right)$	$D_{99\%}\left(\%\right)$	$D_{100\%}\left(\%\right)$
A	AVG	99.5(−0.5)	98.6(0.2)	93.1(−1.8)	99.9 (−0.1)	95.5 (−3.0)	76.7 (−18.2)
	OVR	98.4(−1.6)	96.2(−1.7)	93.2(−2.0)	98.2 (−1.8)	97.7 (−0.2)	83.0 (−12.2)
	MIP	95.4(−4.6)	93.6(−4.8)	91.0(−4.0)	99.2 (−0.8)	97.2 (−1.2)	93.8 (−1.2)
	mAVG	98.7(−1.3)	98.8(0.4)	93.5(−1.4)	100.8 (0.8)	99.2 (0.8)	95.2 (0.3)
B	AVG	98.1 (−1.9)	97.6 (−1.3)	94.5 (−1.9)	89.2 (−10.8)	76.8 (−22.1)	46.3 (−50.1)
	OVR	98.6 (−1.4)	96.9 (−1.7)	93.6 (−2.3)	99.2 (−0.9)	95.1 (−3.5)	82.1 (−13.8)
	MIP	97.5 (−2.5)	93.9 (−4.0)	90.2 (−5.4)	99.4 (−0.6)	97.7 (−0.2)	95.1 (−0.5)
	mAVG	98.9 (−1.1)	97.4 (−1.5)	94.4 (−2.0)	98.9 (−1.1)	97.4 (−1.5)	94.4 (−2.0)
C	AVG	99.5 (−0.5)	97.3 (−1.0)	93.4 (−1.6)	100.0 (0.0)	97.7 (−0.6)	84.0 (−11.0)
	OVR	98.4 (−1.6)	96.4 (−2.0)	92.3 (−1.7)	98.9 (−1.1)	97.7 (−0.7)	90.6 (−3.4)
	MIP	98.8 (−1.2)	95.9 (−2.2)	89.0 (−4.5)	99.2 (−0.8)	97.9 (−0.7)	92.9 (−1.9)
	mAVG	99.8 (−0.2)	97.1 (−1.2)	94.2 (−0.8)	99.5 (−0.5)	96.9 (−1.4)	92.3 (−2.5)
D	AVG	99.2 (−0.8)	96.6 (−1.2)	92.0 (−1.9)	98.1 (−2.0)	85.0 (−12.8)	59.0 (−34.9)
	OVR	99.1 (−0.9)	96.9 (−1.3)	92.2 (−1.3)	99.3 (−0.8)	96.3 (−1.9)	82.0 (−11.5)
	MIP	99.1 (−0.9)	97.0 (−1.1)	92.1 (−1.8)	99.1 (−0.9)	97.3 (−1.1)	93.3 (−0.6)
	mAVG	99.2 (−0.8)	96.7 (−1.8)	92.2 (−1.9)	98.2 (−1.8)	86.0 (−12.5)	61.0 (−33.1)
E	AVG	99.0 (−1.0)	97.3 (−2.0)	92.4 (−3.9)	92.8 (−7.2)	86.5 (−12.8)	74.6 (−21.8)
	OVR	99.1 (−0.9)	97.8 (−0.4)	95.4 (−1.0)	99.8 (−0.25)	92.9 (−5.4)	79.8 (−16.6)
	MIP	94.8 (−5.2)	93.1 (−5.4)	90.5 (−5.9)	98.9 (−1.1)	97.4 (−1.1)	95.5 (−0.8)
	mAVG	98.7 (−1.3)	97.2 (−1.2)	95.4 (−1.4)	100.0 (0.0)	98.0 (−0.4)	96.1 (−0.7)
F	AVG	99.1 (−0.9)	97.4 (2.4)	93.6 (−1.1)	99.3 (−0.7)	98.0 (3.0)	92.4 (−2.3)
	OVR	98.7 (−1.3)	97.1 (1.4)	92.9 (−1.6)	99.1 (−1.0)	98.0 (2.3)	94.7 (0.3)
	MIP	99.0 (−1.0)	96.9 (1.1)	92.4 (−2.4)	99.7 (−0.3)	98.0 (2.3)	94.3 (−0.5)
	mAVG	99.4 (−0.6)	98.2 (−0.3)	94.3 (−1.8)	99.4 (−0.6)	98.5 (−0.1)	95.9 (−0.2)
G	AVG	100.1 (0.1)	97.6 (−0.0)	92.5 (−2.3)	99.6 (−0.4)	96.7 (−0.9)	68.0 (−26.8)
	OVR	100.0 (0.0)	97.5 (−0.9)	92.9 (−2.0)	99.6 (−0.4)	96.3 (−2.1)	88.5 (−6.4)
	MIP	99.8 (−0.2)	97.3 (−0.2)	92.8 (−1.7)	99.9 (−0.1)	97.3 (−0.1)	93.9 (−0.6)
	mAVG	99.8 (−0.2)	97.3 (−1.2)	93.7 (−1.4)	99.6 (−0.4)	97.1 (‐14)	86.0 (−9.1)

Note: $D_{x\%}=$ the minimal dose for covering x% of target volume.

#### B.2 MIP plan

The MIP approach also showed a systematic change in target dose, but its trend was opposite to that in the AVG approach. The isodose surface was shifted to the distal direction of the target volume which induced underdosage at the proximal side of the target volume (Fig. 4(c)). Moreover, the shift was distinct in inspiratory phases, instead of expiratory phases shown in the AV G plans (Figs. 3(a)) and (3b). Because the tissue density is overestimated in MIP CT, the distal range in MIP plan is much increased than that in the AV G plan. This increased distal range is excessive for less dense inspiratory phases, but could be adaptive for dense expiratory phases. As can be seen in other patients, the MIP approach is generally not successful at inspiration phase; the target dose change exceeded the acceptable range in five of seven patients, except for patients D and G, with magnitudes ranging from −1.7%– −5.9% for $D_{100\%}$, 1.1%– −5.4% for $D_{99\%}$, and −0.2%– −5.2% for $D_{95\%}$ at full inspiration phase (see Table 3). On the other hand, the dose variations on the end‐of‐expiration CT, whose CT densities are very close to those in MIP CT, were acceptable in all patients, except for patient F, in whom $D_{99\%}$ exceeded the tolerance, but the excess was only 0.3% (see Table 3).

#### B.3 OVR plan

The respiration‐induced dose changes observed using the OVR approach were very similar to those obtained using the AVG strategy because the two approaches were basically the same, except for the density correction of PTV in the OVR approach. This simple density correction in the OVR approach, however, improved substantially, but not completely, the target dose at expiratory phases. For example, as shown in Figs. 4(a) and 4(b), the clear underdosage yielded with the AVG strategy for patient A (Fig. 4(a)) was mostly removed when applying the OVR approach (Fig. 4(b)). However, as can be seen in Fig. 4(b), small PTV volume at distal side of PTV was still underdosed, indicating that the OVR approach did not fully improve the limitation of the AVG strategy in the case of patient A. Similar results were observed in the other six patients (see Table 3). In particular, the OVR plans greatly improved $D_{100\%}$ relative to those in the AVG plans in all seven patients by 2.1% (patient F) to 36.3% (patient B), but only one patient (patient E) case fully met the criteria for the change in the target dose (within ±2%). It is notable that the density override in the present study may overcorrect the gross tumor motion because the density override was applied to PTV including extra planning margin besides the margin for gross tumor motion. However, our results showed that even this overcorrection was not enough for the present liver patients, implying the basic limitation of the OVR approach.

#### B.4 AVG plan with field‐specific margins

As described above, the AVG plans well covered the proximal sides of target volume, while MIP plans did the distal sides of target volume through the entire respiration phases in all the selected patients. Therefore, it is expectable that adequate target coverage could be achieved if the SOBP width is modified to have the same proximal position with the AVG plan and the same distal position with the MIP plan. In this sense, we added the distal SOBP margin to each field in the AVG plan for each patient by the range difference between MIP and AVG plans (see ‘treatment plan’ in Methods and Materials section above). This modified AVG (mAVG) plan was indeed very successful throughout the entire respiration phases. The target coverages were fully acceptable at the full inspiration phases in all the selected patients, and those at the end‐of‐expiration phases were fully acceptable in four of seven patients (see Table 3). In addition, even in other three patients, only the $D_{100\%}$ was changed over tolerable range, while changes in $D_{99\%}$ and $D_{95\%}$ were fully acceptable, except for those in patient D. This indicated that the mAVG plan with additions of distal margins was very effective in compensating the respiration‐induced internal density change.

### C. Four‐dimensional (4d) dose

**Figure 5:**
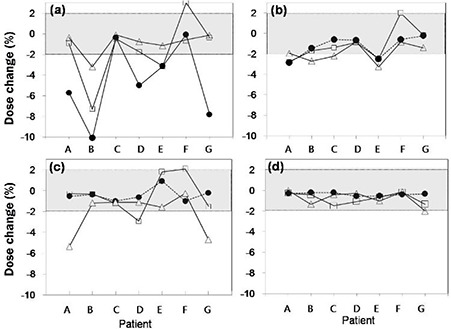
Target dose differences between 4D dose and 3D apparent dose in (a) AVG, (b) MIP, (c) OVR, and (d) mAVG plans, where the filled circles, open boxes, and open triangles represent the difference in $D_{100\%}$, $D_{99\%}$, and $D_{95\%}$, respectively, and the connecting lines are guides for eyes. The acceptable range for the target dose change (±2%) is indicated by gray color‐filled box in each figure.

**Figure 6:**
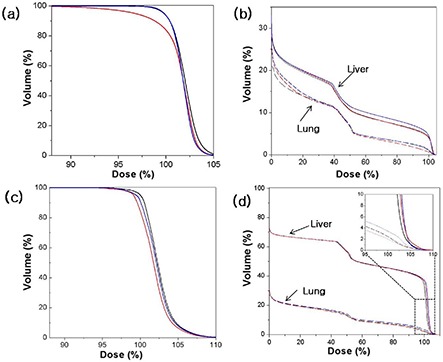
Dose‐volume histograms for PTV ((a) and (c)) and for normal organs ((b) and (d)) of patient B ((a) and (b)) and for patient D ((c) and (d)). In each figure the black, blue, and red colors represent the results from 3D AVG, 4D mAVG, and 4D AVG plans, respectively. In (b) and (d), the dose‐volume relations for liver and lungs are represented by solid and dashed lines, respectively.

In order to evaluate the full effectiveness of these 3D plans, we composed the 3D dose distributions calculated on the 10 respiration CT scans onto the AVG CT scan for each patient^(^ ^15^ ^,^ ^16^ ^)^ (i.e., a 4D dose calculation), where all phases in a respiration cycle were equally weighted. A comparison of dose distribution changes between the 4D and 3D plans are displayed in Fig. 5, which shows the mAVG plans were fully successful in all seven patients with the changes in the target dose indices ($D_{95\%}$, $D_{99\%}$, and $D_{100\%}$) less than 2%. In sharp contrast, only one, three, and four of seven patients were fully acceptable with the AVG, MIP, and OVR plans, respectively. In particular, the AVG plan for patient G and the MIP plan for patient E yielded significantly lower target doses, as all the target‐dose indices for $D_{95\%}$, $D_{99\%}$, and $D_{100\%}$ were out of the ±2% limit. Thus, it was clear that the mAVG strategy was the most effective to produce adequate 4D dose distribution, which might be closer to actual dose distribution. The dose‐volume histograms (DVH) for patients B and D (Fig. 6) further supported the better effectiveness of the mAVG approach, in that the mAVG plans for these patients improved the target coverages in 4D dose relative to those obtained using the AVG approach, and yielded the closer target coverages than those predicted by the ordinary 3D AVG plans. Furthermore, these improvements in target coverages with the mAVG approach did not critically increase the unnecessary dose on surrounding normal organs. For example, the 4D mAVG approach only increased the volumes of normal liver and lung receiving at least 80% of the prescribed dose by 1.5% and 0.5%, respectively, in patient B, and by 0.5% and 0.2%, respectively, in patient D, compared with the AVG approach.

## IV. DISCUSSION

We have investigated the respiration‐induced changes in radiation dose during proton therapy using 4D CT image sets of seven selected liver patients. We found that 3D proton planning methods using a single CT scan did not fully guarantee the adequate delivery of target doses during breathing motion. This was due mainly to the fact that a single planning CT scan cannot exactly reflect the respiration‐induced variations in the tissue‐density distribution, which changed the effective depths of proton beams to reach the targeted positions. We assumed that overall change in the effective depth can be represented by the position differences of SOBP between AVG and MIP plans. This assumption is practically reasonable, since the AVG and MIP plans yielded similar dose distributions predicted by 3D plans at the full‐inspiratory and end‐of‐expiratory phases, respectively. Based on this assumption, we calculated the respiration‐induced changes in effective depth to the targeted position which ranged from 0.03−0.95 cm in the seven patients. This magnitude of effective depth change might be trivial in photon therapy since the resultant change in dose may be not higher than 2%. This magnitude of depth change, however, could be critical in particle therapy, including proton therapy, because the radiation dose is drastically changed with the depth change near the distal side of SOBP. Therefore, particle therapy may involve the potential risk of geometric misses, particularly when treating mobile tumors. Such risk of geometric misses can be seen in the clinical report for lung cancer with carbon‐ion therapy, where marginal recurrence at field edges amounted to as many as ∼32%.^(^ ^23^ ^)^

Several correction methods to avoid the geometric misses of mobile tumors have been proposed,^(^ ^15^ ^–^ ^18^ ^)^ which could be grouped into density correction^(^ ^15^ ^)^ and smearing methods.^(^ ^16^ ^–^ ^18^ ^)^ The density correction schemes, referred to as the OVR strategy in this study, has been reported to be effective in patients with lung cancer as the method yielded sufficient target coverages and even increases target doses compared to that predicted by 3D plan. In spite of such satisfactory result for lung cancer, this method was only partially successful in liver cancer patients. In lung cancer patients, variations in internal density that influence on the effective depth change originates primarily from the gross tumor motions,^(^ ^16^ ^)^ making density corrections of planned target volume sufficient. In liver cancer patients, however, not only the gross tumor motion but also the surrounding normal tissue motions largely affect to the radiological path length change of proton beam. Therefore, the density‐correction scheme, which only considers the density change of tumor volume,^(^ ^15^ ^,^ ^16^ ^)^ is necessarily limited if breathing motion accompanies with the substantial density changes of surrounding normal tissues, as in our patients. In addition, our results showed that both the AVG and MIP CT scans were not proper to design the proton plan. The MIP CT and AVG CT may exaggerate liver and lung volumes, respectively, as they included motion volumes of liver and lung, respectively. These demonstrate that the density correction to any of motion volumes of normal organs also cannot be the proper solution for correcting the respiration‐induced dose change.

Another method to avoid geometric misses of mobile tumors, called the smearing method,^(^ ^16^ ^–^ ^18^ ^)^ smoothen the compensator curvature by replacing its ordinary thickness at a certain point with the minimum thickness within a radius of a given smearing margin, resulting in a broadening of prescribed dose surface. This method, however, also has a limitation because it cannot correct the change in the distal range or the distal edge of SOBP, but simply broadens the isodose surface with respect to the distal end position of proton beam.

Therefore, it is necessary to include an extra margin to extend the SOBP width taking into account the density variations during breathing motion. The issue is, how to obtain the optimal margin, which cannot be determined from any of the previous methods. Furthermore, unlike other planning margins, this extra planning margin should be set for each proton field because the required margin is specific to each field orientation and path length. Our method described in this work provided relatively easy and reasonable way to determine the optimal SOBP margin for each proton field. Our approach basically widens SOBP width to ensure adequate target coverage under respiration motion, suggesting it may not be optimal for normal tissue sparing. However, the unnecessarily increased high doses with addition of extra field margins were significantly blurred out during respiration. Thus, the normal tissue dose was not significantly increased with the mAVG strategy, as shown in Fig. 6.

Based on the results, we suggest a new work flow for 3D proton planning for mobile liver cancers. First, a 3D plan with desired apparent dose distribution must be prepared on AV G CT scan. Next, another 3D plan is constructed on a MIP CT scan, in which only the distal range and SOBP width for each field can be altered to achieve the same apparent dose distribution as the AV G plan, with other planning parameters kept identical to those in the AVG plan. Finally, the 3D AVG plan is recalculated on the AVG CT after adding the extra margin to each field by using the difference in distal ranges between AVG and MIP plans. This work flow is time‐consuming, since it requires extra dose calculations on multiple CT scans. However, in our experience, most of planning time is spent for determining the proper field alignments and related field parameters, while 3D dose calculation under given conditions takes only a few minutes. Because our planning strategy requires only two to three additional 3D dose calculations, the extra calculation time generally does not exceed 10 minutes, which might be acceptable in practice.

## V. CONCLUSIONS

Four different proton planning strategies for mobile liver cancer, the proton planning based on AVG CT (AVG), MIP CT (MIP), AVG CT with density overridden of planned target volume (OVR), and AV G CT with addition of field‐specific proton margin (mAVG) were investigated. The AV G and MIP strategies were only successful in inspiratory and expiratory phases, respectively. These limitations for the AVG and MIP plans were mainly due to underestimation (AVG) and overestimation (MIP) of respiration‐induced changes in internal densities in AV G and MIP CT, respectively. It was found that the OVR approach could partially compensate the limitation of the AVG approach because the density change with tumor motion was further included in this approach beyond the simple AVG approach. However, there was still serious limitation because the density changes associated with liver motion was also not considered in the OVR approach. Among the planning strategies examined in the study, only the mAVG planning strategy was fully successful for the every phase in respiration cycle because the underestimated beam range of each proton field in the AVG plan was fully complemented by the field‐specific proton margin defined as the difference of proton‐bean ranges in between MIP and AV G plans. Furthermore, this increment of distal range with a field‐specific proton margin did not critically raise the dose to normal tissue. These results demonstrated that our new planning strategy with field‐specific proton margins could effectively and efficiently raise a planning quality for mobile liver cancer.

## ACKNOWLEDGMENTS

This study was supported by a grant from the National Research and Development Program for Cancer Control, Ministry for Health and Welfare, Republic of Korea (1110600‐2).
